# Metagenome-assembled genomes from microbiomes fermenting dairy coproducts

**DOI:** 10.1128/mra.00173-24

**Published:** 2024-05-31

**Authors:** Kevin A. Walters, Kevin S. Myers, Timothy J. Donohue, Daniel R. Noguera

**Affiliations:** 1 Great Lakes Bioenergy Research Center, University of Wisconsin-Madison, Madison, Wisconsin, USA; 2 Wisconsin Energy Institute, University of Wisconsin-Madison, Madison, Wisconsin, USA; 3 Department of Bacteriology, University of Wisconsin-Madison, Madison, Wisconsin, USA; 4 Department of Civil and Environmental Engineering, University of Wisconsin-Madison, Madison, Wisconsin, USA; Montana State University, Bozeman, Montana, USA

**Keywords:** microbiome, fermentation, dairy

## Abstract

To advance knowledge of microbial communities capable of fermenting agro-industrial residues into value-added products, we report metagenomes of microbial communities from six anaerobic bioreactors that were fed a mixture of ultra-filtered milk permeate and cottage cheese acid whey. These metagenomes produced 122 metagenome-assembled genomes that represent 34 distinct taxa.

## ANNOUNCEMENT

Microbial communities have the potential to increase the value of agro-industrial residues by fermentation. Metagenomes reported here originate from two sets of anaerobic bioreactors and their inoculant cultures. In each experiment, three chemostats were operated anaerobically, at pH 5.5, 6-day hydraulic retention time, and either 35°C or 50°C. They were fed a 1:1 mixture of ultra-filtered milk permeate and cottage cheese acid whey and inoculated with the culture from an acid-phase digester at the local wastewater treatment plant (Madison, WI, USA) that was enriched in a similarly operated bioreactor for 23 days at 35°C. For each experiment, DNA was extracted, in duplicate, from the inoculant culture and from each bioreactor with the DNeasy PowerSoil Pro kit (Qiagen, Hilden, Germany) following the manufacturer’s protocol. A total of 16 samples containing 1,500 ng DNA were submitted to the Joint Genome Institute (JGI) (Berkeley, USA) for sequencing, assembly, and binning, using the JGI metagenomic workflow (1). Default software parameters were used unless noted. Processing and sequencing of DNA included shearing to 300 bp fragments using a Covaris LE220 (Woburn, USA) and TotalPure NGS beads (Omega Biotek, Atlanta, USA), end repair, A-tailing, ligation of adapters using the Kapa-HyperPrep kit (Roche, USA), and sequencing of paired-end 2 × 150 bp reads using a NovaSeq S4 platform (Illumina, San Diego, USA) resulting in libraries containing 189 to 648 million 150 bp reads. Read processing and assembly included filtering, trimming, and removal of contamination using BBDuk (v38.99) and BBMap (v38.86) (2), error correction using bbcms (v38.86) (mincount=2, highcountfraction=0.6) (2), assembly into contigs with metaSPAdes (v3.15.2) (“spades.py -m 2000 --only-assembler -k 33,55,77,99,127 --meta”) (3) discarding contigs below 200 bp, and mapping the filtered reads onto the contigs with BBMap (v38.86) (“bbmap.sh build=1 fastareadlen=500 interleaved=true ambiguous=random”) (2) to determine coverage for binning purposes. Contigs were binned into metagenome-assembled genomes (MAGs) using MetaBAT (v2:2.15) (4) following the JGI IMG Annotation Pipeline (v5.1.17) (1).

Following JGI processing, MAGs were refined by identifying and removing contaminant contigs with ProDeGe (v2.3) (5) and custom scripts (“run.GC.sh” and “Calculating_TF_Correlations.R”) based on tetranucleotide frequency (6). CheckM (v1.2.2) (7) was used to obtain quality statistics for all MAGs, and MAGs under 75% completeness were discarded. Retained refined MAGs were annotated by NCBI using the Prokaryotic Genome Annotation Pipeline (v6.6) (8). MAGs from all samples were pooled and dereplicated using dRep (v3.4.5) (“dereplicate –conW 0.5 –N50W 5”) (9), whereby near-identical MAGs were clustered, and representative MAGs for each cluster were selected based on quality (Table 1). GTDB-Tk (v2.1.1, database release 214) (10) was used to assign taxonomy to representative MAGs (“gtdbtk identify”) based on concatenated bacterial marker genes (Bac120). This announcement reports 122 annotated medium-quality and high-quality MAGs, grouped into 34 dereplicated clusters representing distinct taxa (Table 1). These data advance our metagenome-based knowledge of agro-industrial residue and waste bioconverting microbiomes (6, 11–20).

**TABLE 1:** MAG statistics and genome accession numbers

Strain name^*a*^	Code^*b*^	Sample of origin^*c*^	ANIm^*d*^	dRep score^*e*^	GTDB-Tk classification	Reference genome^*f*^	ANIm^*g*^	Completeness (%)	Contamination (%)	MAG size (Mbp)	No. of c ontigs	*N*_50_ (Mbp)	%GC	NCBI genome accession number^*h*^	SRA accession number^*i*^	Relative abundance (%)^*j*^	Coverage^*k*^
UW_AWMP_ACET1_1	ACET1	A2_r1		125	d__Bacteria;p__Pseudomonadota;c__Alphaproteobacteria;o__Acetobacterales;f__Acetobacteraceae;g__Acetobacter;s__Acetobacter sp012517935	GCA_012517935.1	0.9580	100	0.25	2.794	28	0.121	53.7	GCA_036452275.1	SRR26909978	<1	33
UW_AWMP_ACET1_2		A1_r1	0.9993	106				79.1	0	2.294	18	0.213	54.0	GCA_036452255.1	SRR26909966	<1	174
UW_AWMP_ACUT1_1	ACUT1	A3_r2		106	d__Bacteria;p__Bacillota_A;c__Clostridia;o__Oscillospirales;f__Acutalibacteraceae;g__Caproicibacterium;s__Caproicibacterium sp022483045	GCA_022483045.1	0.9999	82.55	0	1.599	22	0.086	51.3	GCA_036454745.1	SRR26910051	<1	15
UW_AWMP_ACUT2_1	ACUT2	Series2_r2		103	d__Bacteria;p__Bacillota_A;c__Clostridia;o__Oscillospirales;f__Acutalibacteraceae;g__Caproicibacterium;s__Caproicibacterium sp902809935	GCA_902809935.1	0.9948	82.7	0.34	1.580	82	0.023	42.7	GCA_036454725.1	SRR26909984	<1	21
UW_AWMP_ATO1_1	ATO1	A2_r2		124	d__Bacteria;p__Actinomycetota;c__Coriobacteriia;o__Coriobacteriales;f__Atopobiaceae;g__Tractidigestivibacter;s__Tractidigestivibacter scatoligenes	GCF_001494635.1	0.9565	98.39	0	2.241	40	0.141	62.8	GCA_036454705.1	SRR26909981	<1	158
UW_AWMP_ATO1_2		A2_r1	0.9990	114				90.23	0	2.062	59	0.048	62.5	GCA_036454685.1	SRR26909978	<1	92
UW_AWMP_ATO2_1	ATO2	Series2_r1		121	d__Bacteria;p__Actinomycetota;c__Coriobacteriia;o__Coriobacteriales;f__Atopobiaceae;g__UBA7741;s__	NA	NA	98.39	0.27	2.092	63	0.045	69.7	GCA_036454645.1	SRR26910046	<1	226
UW_AWMP_ATO3_1	ATO3	B1_r1		118	d__Bacteria;p__Actinomycetota;c__Coriobacteriia;o__Coriobacteriales;f__Atopobiaceae;g__UBA7748;s__UBA7748 sp900314535	GCA_900314535.1	0.9723	95.97	0.81	2.022	69	0.035	60.2	GCA_036454665.1	SRR26909979	37	12,486
UW_AWMP_ATO3_2		B1_r2	1.0000	117				94.35	0.81	1.992	68	0.035	60.2	GCA_036454625.1	SRR26909986	37	16,419
UW_AWMP_ATO3_3		Series1_r2	0.9780	116				92.74	0.27	1.679	62	0.035	60.5	GCA_036454605.1	SRR26909968	9	3,877
UW_AWMP_ATO3_4		Series1_r1	0.9793	115				91.94	0.27	1.653	66	0.032	60.5	GCA_036454585.1	SRR26909967	10	3,372
UW_AWMP_ATO3_5		A2_r2	0.9746	101				79.03	0.9	1.578	80	0.024	60.4	GCA_036454565.1	SRR26909981	<1	187
UW_AWMP_ATO4_1	ATO4	A1_r1		107	d__Bacteria;p__Actinomycetota;c__Coriobacteriia;o__Coriobacteriales;f__Atopobiaceae;g__Olegusella;s__Olegusella sp002407925	GCA_002407925.1	0.9865	80.65	0	1.498	8	0.253	55.5	GCA_036454505.1	SRR26909966	12	2,610
UW_AWMP_BACIL1_1	BACIL1	B2_r2		115	d__Bacteria;p__Bacillota;c__Bacilli;o__Bacillales;f__Bacillaceae_C;g__Weizmannia;s__Weizmannia coagulans	GCF_000290615.1	0.9832	91.61	0	2.447	70	0.049	47.9	GCA_036454485.1	SRR26909982	24	3,817
UW_AWMP_BACIL1_2		B2_r1	0.9999	111				87.57	0	2.36	69	0.046	47.7	GCA_036454545.1	SRR26909987	23	5,522
UW_AWMP_BACIL1_3		B3_r1	0.9525	110				87.22	0.55	2.432	92	0.032	47.5	GCA_036454525.1	SRR26909985	11	1,343
UW_AWMP_BACIL1_4		B3_r2	0.9510	110				86.76	0	2.471	90	0.036	47.4	GCA_036454465.1	SRR26910050	10	2,698
UW_AWMP_BIF1_1	BIF1	B3_r2		130	d__Bacteria;p__Actinomycetota;c__Actinomycetia;o__Actinomycetales;f__Bifidobacteriaceae;g__Bifidobacterium;s__Bifidobacterium mongoliense	GCF_000741285.1	0.9757	99.73	1.14	2.109	7	1.312	63.2	GCA_036454425.1	SRR26910050	3	752
UW_AWMP_BIF1_2		B1_r2	1.0000	130				99.73	1.14	2.105	9	1.259	63.2	GCA_036454285.1	SRR26909986	<1	383
UW_AWMP_BIF1_3		A3_r1	1.0000	130				99.73	1.14	2.105	8	1.259	63.2	GCA_036454265.1	SRR26909983	3	640
UW_AWMP_BIF1_4		B2_r2	1.0000	130				99.73	1.14	2.112	10	1.154	63.2	GCA_036454305.1	SRR26909982	2	345
UW_AWMP_BIF1_5		A3_r2	1.0000	130				99.73	1.14	2.11	10	1.154	63.2	GCA_036454225.1	SRR26910051	3	1,327
UW_AWMP_BIF1_6		A1_r1	1.0000	130				99.73	1.14	2.07	9	1.138	63.3	GCA_036454245.1	SRR26909966	1	291
UW_AWMP_BIF1_7		B3_r1	1.0000	127				99.73	1.14	2.108	8	0.371	63.2	GCA_036454205.1	SRR26909985	3	381
UW_AWMP_BIF1_8		A1_r2	1.0000	127				99.73	1.14	2.11	11	0.265	63.2	GCA_036454165.1	SRR26909965	2	391
UW_AWMP_BIF1_9		B1_r1	1.0000	126				99.73	1.14	2.066	12	0.209	63.3	GCA_036454185.1	SRR26909979	<1	279
UW_AWMP_BIF1_10		Series1_r2	1.0000	117				88.82	0.23	1.801	11	0.371	63.3	GCA_036454375.1	SRR26909968	<1	54
UW_AWMP_BIF1_11		Series2_r2	1.0000	115				84.73	0.23	1.679	4	1.259	63.2	GCA_036454405.1	SRR26909984	13	1,762
UW_AWMP_BIF1_12		Series2_r1	1.0000	108				77.91	0.08	1.51	3	1.201	63.3	GCA_036454345.1	SRR26910046	11	4,126
UW_AWMP_BIF1_13		A2_r1	1.0000	108				77.91	0.08	1.506	4	0.937	63.4	GCA_036454365.1	SRR26909978	<1	154
UW_AWMP_BIF1_14		B2_r1	1.0000	107				77.91	0.08	1.523	4	0.805	63.3	GCA_036454325.1	SRR26909987	2	523
UW_AWMP_BIF2_1	BIF2	B3_r1		127	d__Bacteria;p__Actinomycetota;c__Actinomycetia;o__Actinomycetales;f__Bifidobacteriaceae;g__Bifidobacterium;s__Bifidobacterium crudilactis	GCF_000738005.1	0.9749	99.08	2.12	2.462	7	0.509	57.6	GCA_036454125.1	SRR26909985	17	2,029
UW_AWMP_BIF2_2		A2_r2	1.0000	127				99.08	2.12	2.422	7	0.509	57.7	GCA_036454145.1	SRR26909981	15	3,971
UW_AWMP_BIF2_3		A3_r1	1.0000	126				99.08	2.12	2.411	10	0.343	57.7	GCA_036454105.1	SRR26909983	31	5,849
UW_AWMP_BIF2_4		A3_r2	1.0000	117				90.36	1.97	2.192	9	0.343	57.6	GCA_036454085.1	SRR26910051	32	13,341
UW_AWMP_BIF2_5		B1_r1	1.0000	113				84.3	0.98	1.872	4	0.569	57.6	GCA_036454065.1	SRR26909979	9	3,031
UW_AWMP_BIF2_6		Series1_r1	0.9664	107				83.86	1.21	1.852	49	0.052	57.9	GCA_036454025.1	SRR26909967	<1	12
UW_AWMP_BIF2_7		Series1_r2	0.9650	103				80.78	1.67	1.75	50	0.046	57.5	GCA_036454045.1	SRR26909968	<1	12
UW_AWMP_BIF3_1	BIF3	B1_r2		125	d__Bacteria;p__Actinomycetota;c__Actinomycetia;o__Actinomycetales;f__Bifidobacteriaceae;g__Bifidobacterium;s__Bifidobacterium subtile	GCF_000741775.1	0.9894	99.44	2.73	2.748	16	0.283	61.2	GCA_036454005.1	SRR26909986	<1	172
UW_AWMP_BIF3_2		B1_r1	0.9999	123				98.08	2.58	2.551	17	0.201	61.1	GCA_036453985.1	SRR26909979	<1	119
UW_AWMP_BIF4_1	BIF4	A2_r1		125	d__Bacteria;p__Actinomycetota;c__Actinomycetia;o__Actinomycetales;f__Bifidobacteriaceae;g__Bifidobacterium;s__Bifidobacterium thermophilum	GCA_000771265.1	0.9551	100	0.15	2.049	27	0.112	60.5	GCA_036453945.1	SRR26909978	<1	20
UW_AWMP_BIF4_2		A2_r2	0.9994	108				80	0.15	1.47	6	0.351	60.5	GCA_036453925.1	SRR26909981	<1	26
UW_AWMP_BIF5_1	BIF5	A1_r2		123	d__Bacteria;p__Actinomycetota;c__Actinomycetia;o__Actinomycetales;f__Bifidobacteriaceae;g__Bifidobacterium;s__Bifidobacterium sp022649135	GCA_022649135.1	0.9934	98.31	1.53	2.45	41	0.095	61.5	GCA_036453965.1	SRR26909965	<1	113
UW_AWMP_BIF5_2		A1_r1	0.9997	121				97.28	1.38	2.374	46	0.065	61.7	GCA_036453885.1	SRR26909966	<1	89
UW_AWMP_COR1_1	COR1	Series1_r1		120	d__Bacteria;p__Actinomycetota;c__Coriobacteriia;o__Coriobacteriales;f__UMGS124;g__CAIFEU01;s__CAIFEU01 sp903789505	GCA_903789505.1	0.9735	95.16	1.41	2.881	38	0.128	69.5	GCA_036453905.1	SRR26909967	<1	85
UW_AWMP_COR1_2		Series1_r2	0.9998	119				93.55	1.49	3.054	37	0.128	69.4	GCA_036453865.1	SRR26909968	<1	100
UW_AWMP_EGG1_1	EGG1	Series2_r2		121	d__Bacteria;p__Actinomycetota;c__Coriobacteriia;o__Coriobacteriales;f__Eggerthellaceae;g__CAIFEB01;s__CAIFEB01 sp903789375	GCA_903789375.1	0.9674	97.9	0	2.264	63	0.044	64.9	GCA_036453845.1	SRR26909984	<1	18
UW_AWMP_EGG1_2		Series2_r1	0.9985	120				95.9	1.7	2.21	64	0.042	64.9	GCA_036453785.1	SRR26910046	<1	46
UW_AWMP_ENTER1_1	ENTER1	Series2_r1		130	d__Bacteria;p__Pseudomonadota;c__Gammaproteobacteria;o__Enterobacterales;f__Enterobacteriaceae;g__Rahnella;s__Rahnella inusitata	GCF_003263515.1	0.9896	98.03	0.08	5.063	10	2.625	53.1	GCA_036453825.1	SRR26910046	<1	365
UW_AWMP_ENTER1_2		B1_r2	1.0000	130				98.03	0.08	5.063	10	2.625	53.1	GCA_036453645.1	SRR26909986	1	498
UW_AWMP_ENTER1_3		B2_r1	1.0000	130				97.87	0.08	5.063	11	2.625	53.1	GCA_036453605.1	SRR26909987	<1	135
UW_AWMP_ENTER1_4		Series2_r2	1.0000	130				97.87	0.08	5.063	10	2.625	53.1	GCA_036453565.1	SRR26909984	<1	130
UW_AWMP_ENTER1_5		B2_r2	1.0000	128				98.03	0.08	5.064	11	1.188	53.1	GCA_036453585.1	SRR26909982	<1	95
UW_AWMP_ENTER1_6		A1_r2	1.0000	128				97.87	0.08	5.063	11	1.188	53.1	GCA_036453525.1	SRR26909965	<1	88
UW_AWMP_ENTER1_7		B1_r1	1.0000	128				97.87	0.08	5.067	12	1.114	53.1	GCA_036453545.1	SRR26909979	1	382
UW_AWMP_ENTER1_8		A1_r1	1.0000	127				97.87	0.08	5.034	12	0.634	53.1	GCA_036453505.1	SRR26909966	<1	57
UW_AWMP_ENTER1_9		B3_r2	0.9999	126				98.2	0.08	5.059	20	0.377	53.1	GCA_036453485.1	SRR26910050	<1	30
UW_AWMP_ENTER1_10		A3_r2	1.0000	126				97.87	0.08	5.04	17	0.417	53.1	GCA_036453805.1	SRR26910051	<1	49
UW_AWMP_ENTER1_11		A3_r1	0.9999	125				98.52	0.08	5.025	30	0.245	53.1	GCA_036453765.1	SRR26909983	<1	24
UW_AWMP_ENTER1_12		Series1_r2	1.0000	125				97.7	0.08	5.042	21	0.35	53.1	GCA_036453715.1	SRR26909968	<1	36
UW_AWMP_ENTER1_13		A2_r2	1.0000	125				98.2	0.08	5.019	28	0.278	53.2	GCA_036453725.1	SRR26909981	<1	23
UW_AWMP_ENTER1_14		Series1_r1	1.0000	125				97.87	0.19	5.045	28	0.255	53.1	GCA_036453695.1	SRR26909967	<1	31
UW_AWMP_ENTER1_15		B3_r1	0.9998	123				97.87	0.08	4.819	58	0.13	53.4	GCA_036453665.1	SRR26909985	<1	16
UW_AWMP_ENTER1_16		A2_r1	1.0000	123				97.7	0.08	4.84	76	0.095	53.4	GCA_036453625.1	SRR26909978	<1	15
UW_AWMP_ERY1_1	ERY1	A2_r2		120	d__Bacteria;p__Bacillota;c__Bacilli;o__Erysipelotrichales;f__Erysipelotrichaceae;g__Bulleidia;s__	NA	NA	96.19	0.45	2.114	59	0.05	47.9	GCA_036453465.1	SRR26909981	<1	35
UW_AWMP_ERY1_2		A2_r1	0.9994	111				89.52	0.45	1.825	100	0.019	48.2	GCA_036453445.1	SRR26909978	<1	14
UW_AWMP_ERY2_1	ERY2	Series1_r1		115	d__Bacteria;p__Bacillota;c__Bacilli;o__Erysipelotrichales;f__Erysipelotrichaceae;g__Bulleidia;s__Bulleidia sp900319505	GCA_900319505.1	0.9523	92.52	0.32	1.691	66	0.03	47.9	GCA_036453425.1	SRR26909967	17	6,142
UW_AWMP_ERY2_2		Series1_r2	0.9995	98				75.37	0.32	1.462	54	0.031	47.9	GCA_036453385.1	SRR26909968	16	7,190
UW_AWMP_LAC1_1	LAC1	A1_r1		119	d__Bacteria;p__Bacillota;c__Bacilli;o__Lactobacillales;f__Lactobacillaceae;g__Lactobacillus;s__Lactobacillus delbrueckii	GCF_001433875.1	0.9759	95.78	0	1.797	54	0.046	50.1	GCA_036453405.1	SRR26909966	9	2,072
UW_AWMP_LAC1_2		B1_r1	0.9995	116				93.18	0	1.573	56	0.031	50.6	GCA_036455785.1	SRR26909979	<1	107
UW_AWMP_LAC1_3		A2_r2	0.9994	115				93.18	0	1.531	64	0.025	50.8	GCA_036455715.1	SRR26909981	<1	15
UW_AWMP_LAC1_4		B3_r2	0.9830	114				88.85	0	1.597	29	0.088	50.6	GCA_036455765.1	SRR26910050	5	1,304
UW_AWMP_LAC1_5		B1_r2	0.9995	114				90.58	0	1.712	59	0.038	50.1	GCA_036455695.1	SRR26909986	<1	166
UW_AWMP_LAC2_1	LAC2	A1_r2		102	d__Bacteria;p__Bacillota;c__Bacilli;o__Lactobacillales;f__Lactobacillaceae;g__Lactobacillus;s__Lactobacillus absiana	GCA_017309015.1	0.9782	79.99	0.97	1.392	75	0.019	52.7	GCA_036455685.1	SRR26909965	<1	177
UW_AWMP_LAC2_2		B1_r1	0.9943	101				78.35	0	1.374	42	0.038	52.2	GCA_036455705.1	SRR26909979	4	1,195
UW_AWMP_LAC2_3		B1_r2	0.9941	98				75.11	0	1.351	40	0.039	52.1	GCA_036453365.1	SRR26909986	4	1,588
UW_AWMP_LAC3_1	LAC3	A1_r2		100	d__Bacteria;p__Bacillota;c__Bacilli;o__Lactobacillales;f__Lactobacillaceae;g__Lactobacillus;s__Lactobacillus amylovorus	GCF_002706375.1	0.9756	79.28	0.09	1.343	73	0.022	38.6	GCA_036453345.1	SRR26909965	<1	17
UW_AWMP_LACCAS1_1	LACCAS1	A3_r1		125	d__Bacteria;p__Bacillota;c__Bacilli;o__Lactobacillales;f__Lactobacillaceae;g__Lacticaseibacillus;s__Lacticaseibacillus paracasei	GCF_000829035.1	0.9836	99.46	0	2.788	39	0.102	46.5	GCA_036453305.1	SRR26909983	9	1,765
UW_AWMP_LACCAS1_2		A3_r2	1.0000	124				98.91	0	2.787	38	0.102	46.5	GCA_036453325.1	SRR26910051	8	3,476
UW_AWMP_LACCAS1_3		B1_r1	0.9905	111				87.47	0	2.185	51	0.06	46.6	GCA_036453275.1	SRR26909979	<1	157
UW_AWMP_LACCAS2_1	LACCAS2	B1_r2		107	d__Bacteria;p__Bacillota;c__Bacilli;o__Lactobacillales;f__Lactobacillaceae;g__Lacticaseibacillus;s__Lacticaseibacillus rhamnosus	GCF_900636965.1	0.9749	80.98	0	2.071	23	0.133	46.8	GCA_036453285.1	SRR26909986	<1	62
UW_AWMP_LACCAS2_2		B1_r1	1.0000	101				75.69	0	1.992	23	0.133	46.8	GCA_036453265.1	SRR26909979	<1	45
UW_AWMP_LCO1_1	LCO1	Series2_r2		121	d__Bacteria;p__Bacillota_A;c__Clostridia;o__Lachnospirales;f__Lachnospiraceae;g__Bilifractor;s__Bilifractor sp900319355	GCA_900319355.1	0.9934	97.13	0.32	2.31	40	0.081	52.5	GCA_036453235.1	SRR26909984	<1	30
UW_AWMP_LCO2_1	LCO2	A2_r1		118	d__Bacteria;p__Bacillota_A;c__Clostridia;o__Lachnospirales;f__Lachnospiraceae;g__CAG-791;s__CAG-791 sp900315055	GCA_900315055.1	0.9889	94.91	0.4	2.906	106	0.038	55.9	GCA_036453225.1	SRR26909978	<1	28
UW_AWMP_LCO2_2		A2_r2	0.9999	118				94.91	0.4	2.915	105	0.037	55.9	GCA_036453185.1	SRR26909981	<1	43
UW_AWMP_LCO3_1	LCO3	Series1_r1		116	d__Bacteria;p__Bacillota_A;c__Clostridia;o__Lachnospirales;f__Lachnospiraceae;g__Bilifractor;s__	NA	NA	91.56	1.27	2.395	17	0.174	50.6	GCA_036453165.1	SRR26909967	<1	130
UW_AWMP_LCO4_1	LCO4	A2_r1		114	d__Bacteria;p__Bacillota_A;c__Clostridia;o__Lachnospirales;f__Lachnospiraceae;g__UBA1066;s__	NA	NA	89.81	0	2.176	67	0.054	55.1	GCA_036453195.1	SRR26909978	4	617
UW_AWMP_LCO4_2		A2_r2	0.9999	114				89.81	0	2.162	65	0.054	55	GCA_036453145.1	SRR26909981	4	1,050
UW_AWMP_LCO4_3		Series1_r1	0.9989	113				88.54	0	1.992	40	0.086	55	GCA_036453125.1	SRR26909967	22	7,869
UW_AWMP_LCO4_4		Series1_r2	0.9989	113				88.54	0	1.994	40	0.086	55	GCA_036453075.1	SRR26909968	22	9,531
UW_AWMP_LENLAC1_1	LENLAC1	B1_r2		125	d__Bacteria;p__Bacillota;c__Bacilli;o__Lactobacillales;f__Lactobacillaceae;g__Lentilactobacillus;s__Lentilactobacillus sunkii	GCF_001435575.1	0.966	98.89	0	2.749	25	0.143	42.1	GCA_036453105.1	SRR26909986	<1	21
UW_AWMP_LENLAC1_2		B1_r1	1.0000	124				98.91	0	2.623	45	0.088	42.3	GCA_036453065.1	SRR26909979	<1	15
UW_AWMP_LEUC1_1	LEUC1	B2_r1		128	d__Bacteria;p__Bacillota;c__Bacilli;o__Lactobacillales;f__Lactobacillaceae;g__Leuconostoc;s__Leuconostoc gelidum	GCF_000166715.1	0.9875	99.44	0.19	1.871	8	0.443	36.6	GCA_036453045.1	SRR26909987	<1	150
UW_AWMP_LEUC1_2		B2_r2	1.0000	128				99.44	0.19	1.879	8	0.442	36.6	GCA_036453025.1	SRR26909982	<1	102
UW_AWMP_LEUC1_3		B3_r2	1.0000	128				99.44	0.19	1.878	7	0.442	36.6	GCA_036452995.1	SRR26910050	<1	158
UW_AWMP_LEUC1_4		A3_r1	1.0000	128				99.44	0.19	1.88	6	0.442	36.6	GCA_036452985.1	SRR26909983	<1	80
UW_AWMP_LEUC1_5		B3_r1	0.9999	128				99.44	0.19	1.88	8	0.442	36.6	GCA_036452945.1	SRR26909985	<1	65
UW_AWMP_LEUC1_6		Series2_r1	0.9999	120				90.45	0.19	1.797	4	1.041	36.6	GCA_036452965.1	SRR26910046	<1	101
UW_AWMP_LEUC1_7		A3_r2	0.9999	119				90.45	0.19	1.788	6	0.442	36.6	GCA_036452925.1	SRR26910051	<1	145
UW_AWMP_LEUC1_8		Series2_r2	0.9999	116				90.45	0.19	1.786	17	0.125	36.6	GCA_036452905.1	SRR26909984	<1	32
UW_AWMP_LEUC2_1	LEUC2	A2_r1		125	d__Bacteria;p__Bacillota;c__Bacilli;o__Lactobacillales;f__Lactobacillaceae;g__Leuconostoc;s__Leuconostoc mesenteroides	GCF_000014445.1	0.9903	100	0	1.801	24	0.093	37.7	GCA_036452885.1	SRR26909978	1	186
UW_AWMP_LEUC2_2		A3_r2	0.9995	123				97.88	0	1.595	20	0.103	37.8	GCA_036452855.1	SRR26910051	<1	43
UW_AWMP_LEUC2_3		B2_r2	0.9995	123				97.88	0	1.625	20	0.098	37.9	GCA_036452825.1	SRR26909982	<1	43
UW_AWMP_LEUC2_4		B2_r1	0.9995	123				97.88	0	1.572	17	0.098	37.9	GCA_036452785.1	SRR26909987	<1	68
UW_AWMP_LEUC2_5		A3_r1	1.0000	108				83.6	0	1.294	18	0.087	37.7	GCA_036452815.1	SRR26909983	<1	22
UW_AWMP_PROP1_1	PROP1	A3_r1		126	d__Bacteria;p__Actinomycetota;c__Actinomycetia;o__Propionibacteriales;f__Propionibacteriaceae;g__Acidipropionibacterium;s__Acidipropionibacterium acidipropionici	GCF_001441165.1	0.9883	100	0	3.48	29	0.188	68.9	GCA_036452805.1	SRR26909983	<1	70
UW_AWMP_PROP1_2		A3_r2	1.0000	126				99.34	0	3.482	29	0.178	68.9	GCA_036452765.1	SRR26910051	<1	147
UW_AWMP_PSEU1_1	PSEU1	Series2_r1		98	d__Bacteria;p__Pseudomonadota;c__Gammaproteobacteria;o__Pseudomonadales;f__Pseudomonadaceae;g__Pseudomonas_E;s__Pseudomonas_E helleri	GCF_001043025.1	0.9728	75.79	0	5.159	131	0.053	58.6	GCA_036452715.1	SRR26910046	<1	54
UW_AWMP_STREP1_1	STREP1	A2_r1		127	d__Bacteria;p__Bacillota;c__Bacilli;o__Lactobacillales;f__Streptococcaceae;g__Lactococcus;s__Lactococcus lactis	GCF_900099625.1	0.9846	100	0.28	2.51	20	0.234	34.9	GCA_036452675.1	SRR26909978	2	290
UW_AWMP_STREP1_2		A2_r2	0.9999	127				100	0.28	2.606	21	0.234	35	GCA_036452555.1	SRR26909981	2	563
UW_AWMP_STREP1_3		Series2_r2	0.9999	127				100	0.28	2.552	19	0.234	34.9	GCA_036452575.1	SRR26909984	3	543
UW_AWMP_STREP1_4		Series1_r1	0.9999	127				100	0.28	2.552	19	0.234	34.9	GCA_036452605.1	SRR26909967	<1	331
UW_AWMP_STREP1_5		B3_r1	1.0000	127				100	0.28	2.491	19	0.23	34.9	GCA_036452545.1	SRR26909985	3	355
UW_AWMP_STREP1_6		B3_r2	1.0000	127				100	0.28	2.51	20	0.223	34.9	GCA_036452585.1	SRR26910050	4	941
UW_AWMP_STREP1_7		A1_r1	1.0000	127				100	0.28	2.506	24	0.223	34.9	GCA_036452505.1	SRR26909966	2	509
UW_AWMP_STREP1_8		B2_r1	1.0000	126				99.62	0.28	2.511	23	0.223	34.9	GCA_036452485.1	SRR26909987	7	1,642
UW_AWMP_STREP1_9		A1_r2	1.0000	108				81.51	0.28	1.919	16	0.23	35	GCA_036452465.1	SRR26909965	3	609
UW_AWMP_STREP1_10		Series1_r2	1.0000	108				81.51	0.28	1.937	18	0.23	34.9	GCA_036452705.1	SRR26909968	1	540
UW_AWMP_STREP1_11		Series2_r1	1.0000	108				81.51	0.28	1.938	17	0.23	34.9	GCA_036452685.1	SRR26910046	3	1,296
UW_AWMP_STREP1_12		A3_r2	1.0000	108				81.51	0.28	1.919	16	0.23	35	GCA_036452655.1	SRR26910051	1	603
UW_AWMP_STREP2_1	STREP2	B1_r2		109	d__Bacteria;p__Bacillota;c__Bacilli;o__Lactobacillales;f__Streptococcaceae;g__Lactococcus;s__Lactococcus cremoris	GCF_001591705.1	0.9826	85.85	0.38	1.744	56	0.037	35.7	GCA_036452495.1	SRR26909986	2	1,047
UW_AWMP_STREP2_2		B1_r1	0.9998	97				75.63	0.38	1.589	81	0.021	35.9	GCA_036452445.1	SRR26909979	2	774

^*a*^ Strain name assigned to each MAG. The UW_AWMP prefix stands for University of Wisconsin Acid Whey and Milk Permeate bioreactor. MAGs are clustered during dereplication using dRep (9). Strains with a numerical suffix of _1 are the highest quality dRep representative MAGs for a given cluster; nonrepresentative MAGs in each cluster are assigned the same strain name with sequential numerical suffixes (e.g., _2 and _3), assigned in order of decreasing quality, according to the dRep score.

^*b*^ ACET, *Acetobacteraceae*; ACUT, *Acutalibacteraceae*; ATO, *Atopobiaceae*; BACIL, *Bacillaceae*; BIF, *Bifidobacteriaceae*; COR, *Coriobacteriales*; EGG, *Eggerthellaceae*; ENTER, *Enterobacteriaceae*; ERY, *Erysipelotrichaceae*; LAC, *Lactobacillus*; LACCAS, *Lacticaseibacillus*; LCO, *Lachnospiraceae*; LENLAC, *Lentilactobacillus*; LEUC, *Leuconostoc*; PROP, *Propionibacteriaceae*; PSEU, *Pseudomonadaceae*; STREP, *Streptococcaceae*.

^*c*^ Sample from which a given MAG was derived. A1–A3: samples originating from the three bioreactors operated at 35°C after 140 days of operation; B1–B3: samples originating from the three bioreactors operated at 50°C after 140 days of operation; Series1 and Series2: samples originating from the inoculant. Series1 inoculant was used for bioreactors A1, A2, and B1, while the Series2 inoculant was used for bioreactors A3, B2, and B3. The _r1 and _r2 suffixes denote extraction replicates 1 and 2, respectively.

^*d*^ Average nucleotide identity (ANI) between representative MAG and other MAGs included in the same cluster by dRep.

^*e*^ dRep scoring calculation: (*A* × completeness) − (*B* × contamination) + {*C* × [contamination × (strain heterogeneity/100)]} + [*D* × log(*N*_50_)] + [*E* × log(genome size)] + [*F* × (centrality − S_ANI)], where *A* to *F* were weighted with values 1, 0.5, 1, 5, 0, and 1, respectively. Custom dRep parameters were used to keep continuity with our previous metagenomic projects (15).

^*f*^ NCBI GenBank accession number of the reference genome in GTDB-Tk (10) that is closest to the representative MAG; NA represents MAGs without a closely matched reference genome when using the default minimum alignment fraction of 0.65.

^*g*^ Average nucleotide identity (ANI) between representative MAG and GTDB-Tk reference genome.

^*h*^ NCBI GenBank accession number for each reported MAG.

^*i*^ NCBI SRA accession number for the raw reads of the metagenome for each reported MAG.

^*j*^ Relative abundance calculation: [(mean coverage of MAG of interest)/(sum of mean coverages of each MAG used in mapping)] × (proportion of reads that were mapped to MAGs). These values were determined for each MAG by first using Bowtie2 (v2.4.1; “bowtie2” command) (21) to map BBDuk-filtered reads from each metagenome’s FASTQ file to a concatenated FASTA file of the MAGs generated from that metagenome. Then, resulting SAM files were converted to BAM files and sorted using samtools (v1.10; “samtools view” and “samtools sort” commands) (22). Lastly, CoverM (v0.4.0; “coverm genome” command) (https://wwood.github.io/CoverM/coverm-genome.html) to generate relative abundance statistics from the mapped reads.

^*k*^ Coverage denotes the mean number of aligned reads overlapping each position on the genome using the following equation, where read length equals 150 bp: [(number of reads mapped to a MAG) × (read length)]/(MAG size). These values were determined for each MAG in the same manner as relative abundance, except using the “-m mean” flag when running the “coverm genome” command.

## Data Availability

Raw metagenomic sequence data and annotated MAGs for each sample are available at NCBI GenBank under accession number PRJNA1040840. Links and accession numbers for individual MAGs (NCBI) and raw sequence data (SRA) are presented in Table 1. Custom scripts are available on GitHub (https://github.com/GLBRC/metagenome_analysis).
